# Targeted mutagenesis in mice via an engineered AsCas12f1 system

**DOI:** 10.1007/s00018-023-05100-3

**Published:** 2024-01-28

**Authors:** Peng Fan, Hejun Wang, Feiyu Zhao, Tao Zhang, Jinze Li, Xiaodi Sun, Yongduo Yu, Haoyang Xiong, Liangxue Lai, Tingting Sui

**Affiliations:** 1 State Key Laboratory for Diagnosis and Treatment of Severe Zoonotic Infectious Diseases, Key Laboratory for Zoonosis Research of the Ministry of Education, College of Veterinary Medicine, Jilin University, Changchun, 130062 China; 2 Key Laboratory of Regenerative Biology, Guangzhou Institutes of Biomedicine and Health, Chinese Academy of Sciences, Guangzhou, 510530 Guangdong China

**Keywords:** AsCas12f1, sgRNA engineering, Genome editing efficiency, Mouse

## Abstract

SpCas9 and AsCas12a are widely utilized as genome editing tools in human cells, but their applications are largely limited by their bulky size. Recently, AsCas12f1 protein, with a small size (422 amino acids), has been demonstrated to be capable of cleaving double-stranded DNA protospacer adjacent motif (PAM). However, low editing efficiency and large differences in activity against different genomic loci have been a limitation in its application. Here, we show that engineered AsCas12f1 sgRNA has significantly improved the editing efficiency in human cells and mouse embryos. Moreover, we successfully generated three stable mouse mutant disease models using the engineered CRISPR-AsCas12f1 system in this study. Collectively, our work uncovers the engineered AsCas12f1 system expands mini CRISPR toolbox, providing a remarkable promise for therapeutic applications.

**Supplementary Information:**

The online version contains supplementary material available at 10.1007/s00018-023-05100-3.

## Introduction

Programmable clustered regularly interspaced short palindromic repeats (CRISPR) associated Cas12f endonucleases, also known as Cas14 [1–3]. Cas12f effector proteins, the single-stranded DNA (ssDNA)-specific nucleases, have exhibited abilities to cleave double-stranded DNA (dsDNA) with 5′ T-rich or C-rich protospacer adjacent motifs (PAMs) [4–6]. Up to date, AsCas12f1 (422 aa) [5], Un1Cas12f1 (529 aa) [4, 7], SpaCas12f1 (497 aa) [8], RhCas12f1 (415 aa) [6], and OsCas12f1 (433 aa) [6] have been experimentally validated for programmed DNA cleavage in mammalian cells. However, compared with the well-characterized Cas9 and Cas12a complexes, the CRISPR-Cas12f systems are less potent in cleaving dsDNA and show larger activity variations when targeting different genomic loci [1, 2, 9–11]. Therefore, improving the editing efficiency of CRISPR-Cas12f systems becomes a key point for widespread applications.

The Cas12f from Acidibacillus sulfuroxidans (AsCas12f1), which consists of 422 amino acids, can cleave double-stranded DNA targets with a NTTR (where R represents A or G) PAM [2, 5]. Meanwhile, AsCas12f1 exhibits low but detectable genome editing activity in human cells [12–14]. By engineering the sgRNA, the naturally ineffective CRISPR-Un1Cas12f1 was turned into an efficient genome editing nuclease for mammalian genome regulation [7, 8]. Therefore, AsCas12f1 shows great promise as a miniature genome editing tool.

In this study, we took rational methods to engineer the AsCas12f1 sgRNA scaffold and obtained As-v1 variant. The results of deep sequencing showed that the engineered AsCas12f1 system generated 3.17-fold more efficiently than the wild type (WT) in the human genome. In addition, we tested genome editing in mouse embryos using the optimized version, and successfully generated mouse disease models. Together, the engineered AsCas12f1 system enables robust and faithful gene editing in mammalians.

## Materials and methods

### Animals

All mice mentioned were obtained from the Laboratory Animal Center of Jilin University (Changchun, China). All animal studies were conducted according to experimental practices and standards approved by the Animal Welfare and Research Ethics Committee at Jilin University (SY202302001).

### Plasmid construction

The pCMV-AsCas12f1 plasmid was a kind gift from Quanjiang Ji. All the sgRNA scaffold fragments were synthesized and cloned into pCMV-AsCas12f1 plasmid by Genscript Biotech (Nanjing, China). The Un1Cas12f1 plasmid was obtained from Addgene (#176544).

### Cell culture and transfection

HEK293T and N_2_a cells were cultured in Dulbecco’s modified Eagle’s medium (DMEM) (Meilun Biotechnology Co., Ltd) supplemented with 10% fetal bovine serum (HyClone) and incubated at 37 °C with 5%CO_2_. The cells were seeded into six-well plates and transfected using Hieff Trans™ Liposomal Transfection Reagent (Yeasen, Shanghai, China) according to manufacturer’s instructions [15]. After 72 h of transfection, genomic DNA was collected and used for genotyping. The sequencing of sgRNA and the primers used for genotyping are listed in Table S1 and S2.

### mRNA and gRNA preparation

The pCMV-AsCas12f1 was linearized with XbaI, and transcribed in vitro using the HiScribe™ T7 ARCA mRNA kit (NEB). The mRNA was purified using the RNeasy Mini Kit (Qiagen) according to the manufacturer’s protocol [16]. Cloning of an annealed complementary oligonucleotide pair into a BsaI digested gRNA expression vector (Table S2). The sgRNAs were transcripted using the MAXIscript T7 kit (Ambion) and purified using miRNeasy Mini Kit (Qiagen) in vitro.

### Microinjection of mice zygotes

The protocol used for the microinjection of pronuclear-stage embryos has been described in detail in our previous study [16, 17]. In brief, a mixture of gRNAs (40 ng/μl) and AsCas12f1 (80 ng/μl) was injected into the cytoplasm of pronuclear-stage embryos. The injected mouse zygotes were cultured at 37 °C under 5% CO_2_ in air until the two-cell stage, after which approximately 30–50 injected zygotes were then transferred into the oviducts of the recipient mice.

### Single-embryo PCR amplification and mouse genotyping

The single-embryo PCR amplification and mouse genotyping were performed according to our previous study [17]. All the primers for genotyping are listed in Table S2.

### H&E staining

The skin from WT and KO mice were fixed with 4% paraformaldehyde for 48 h, embedded in paraffin wax, sectioned, mounted on slides, stained with H&E [18, 19] and imaged with a Nikon TS100 microscope.

### Open-field test

The open-field test was used to assess the locomotor and exploratory activity of the mice when they were placed in an unfamiliar environment [20]. The open field was a (l × w × h, 60 cm × 30 cm × 15 cm) box with a non-reflective rectangle base. We defined the center area (30 cm × 15 cm) and the margin area (between the side and 80% of the bottom area, outside of 48 cm × 24 cm). For each testing session, a mouse was placed individually in the center of the arena and allowed to freely explore the environment for 7 min. Travel path were detected by Image J and Animal Tracker 5.6 software [20].

### Novelty-suppressed feeding assay

It consisted of a square base (l × w: 60 cm × 30 cm) with a small pre-weighed food chow pellet (sweets) placed in the center of the arena on a small fixed dish. The mice were individually placed in a clean cage without food 24 h before testing. Feeding was defined as the mouse grabbing the pellet and starting to eat. The mouse was removed after 6 min. The latency for a mouse to approach and eat a new food in a novel environment following an extended period (16–24 h) of food deprivation was used as an index of anxiety-like behavior [21].

### Off-target assay

The potential off-target sites for each target in mice were predicted using an online tool (http://www.rgenome.net/cas-offinder/) [22]. First, select PAM type according to specific CRISPR/Cas-derived RNA-guided endonucleases, including AsCas12f1 (PAM:NTTR, R = A or G); next, select the vertebrate genomes and the corresponding Mus musculus (mm10)—mouse genome; then write crRNA sequences without PAM sequences and select the mismatch number to get potential off-target sites. Finally, click submit. The genomic regions surrounding the off-target site were PCR amplified and then subjected to deep sequencing. Mutations were detected by Hi-TOM analysis (http://121.40.237.174/Hi-TOM/). Hi-TOM analysis is an online tool for high-throughput mutation tracking, which can accurately analyze the mutation percentage of each on- and off-target sites [23]. Off-target sites and primers used to amplify target sequences are listed in Table S3 and S4.

### Statistical analysis

The indel editing efficiencies of AsCas12f1 were determined by deep sequencing. All data are expressed as mean ± s.d. (standard deviation), with at least three individual determinations in all experiments. The data were analyzed with *t* tests using Graphpad prism software 8.0.2. A probability of *p* < 0.05 was considered statistically significant. *p < 0.05, **p < 0.01, ***p < 0.001.

## Results

### Engineered AsCas12f1-associated gRNA with elevated editing efficiency in HEK293T cells

To confirm the editing efficiency of WT CRISPR-AsCas12f1 system, 15 target sites with qualified PAMs were selected in HEK293T cells. The results of indels showed that AsCas12f1 generated low efficient editing, which was difficult to be a mammal genome editing tool (Fig. S1).

**Fig. 1:**
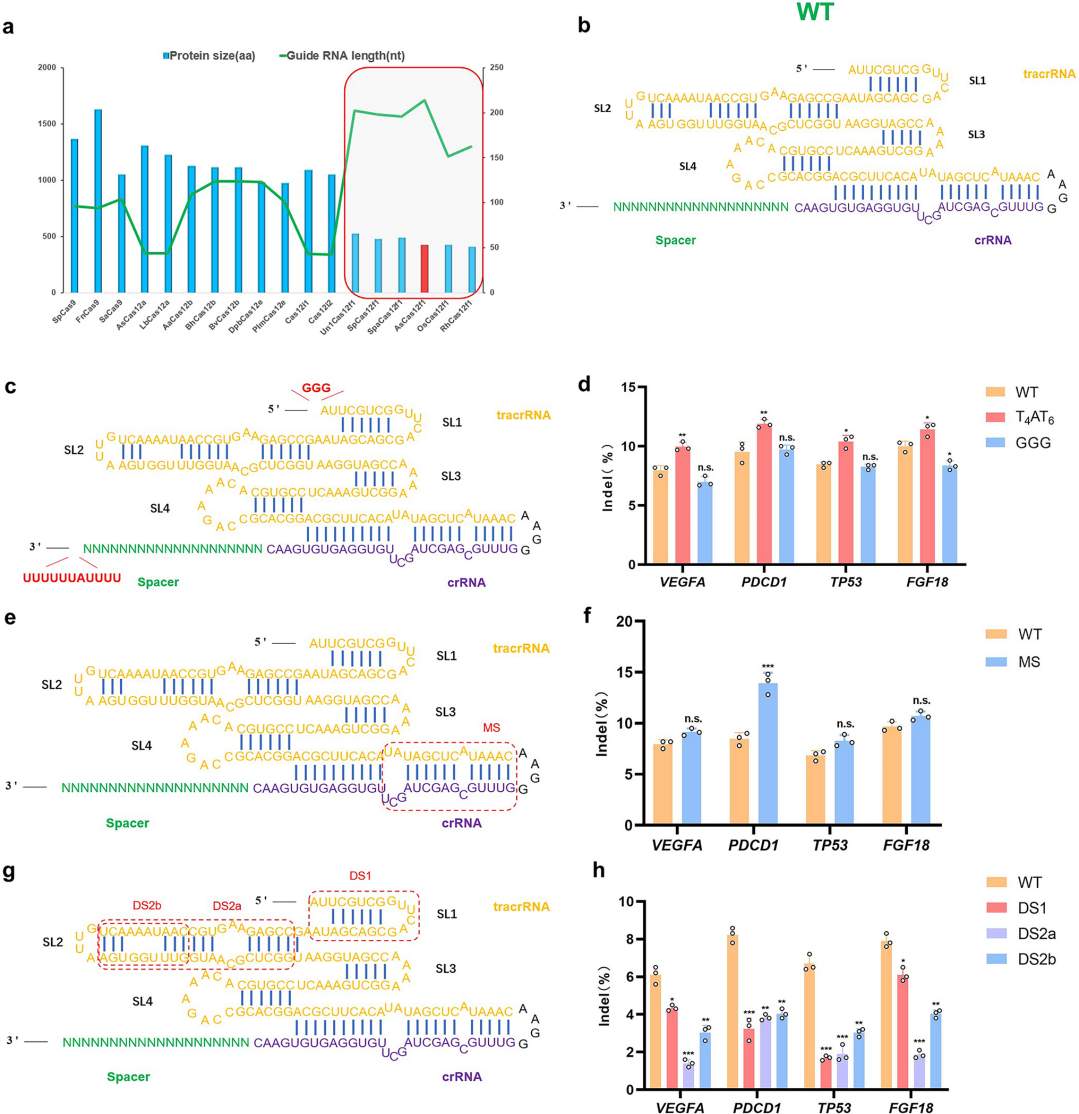
Different gRNA engineering schemes and their mediated indels in human HEK293T cells. **a** Size comparison (aa) of conventional Cas effector proteins (bars) and respective guide RNA length (green line) (nt). The V-F type nucleases are highlighted in the gray area, AsCas12f1 protein is highlighted in the red bar. **b** Structure of the AsCas12f1 WT gRNA consisting of tracrRNA and crRNA. **c** T4AT6/GGG schemes for gRNA engineering are indicated in schematic diagram. T4AT6, TTTTATTTTTT. **d** The indel frequencies of AsCas12f1 usingT4AT6/GGG engineered gRNAs at four different target sites in HEK293T cells. Values and error bars represent mean and s.d. (*n* = 3). **e** MS scheme for gRNA engineering is indicated in schematic diagram. MS, modify site throughout the tracrRNA and crRNA. **f** The indel frequencies of AsCas12f1 using MS engineered gRNA at four different target sites in HEK293T cells. Values and error bars represent mean and s.d. (*n* = 3). **g** DS schemes for gRNA engineering are indicated in schematic diagram. DS, delete site of tracRNA. **h** The indel frequencies of AsCas12f1 using DS engineered gRNA at four different target sites in HEK293T cells. Values and error bars represent mean and s.d. (*n* = 3). *p < 0.05; **p < 0.01; ***p < 0.001

**Fig. 2:**
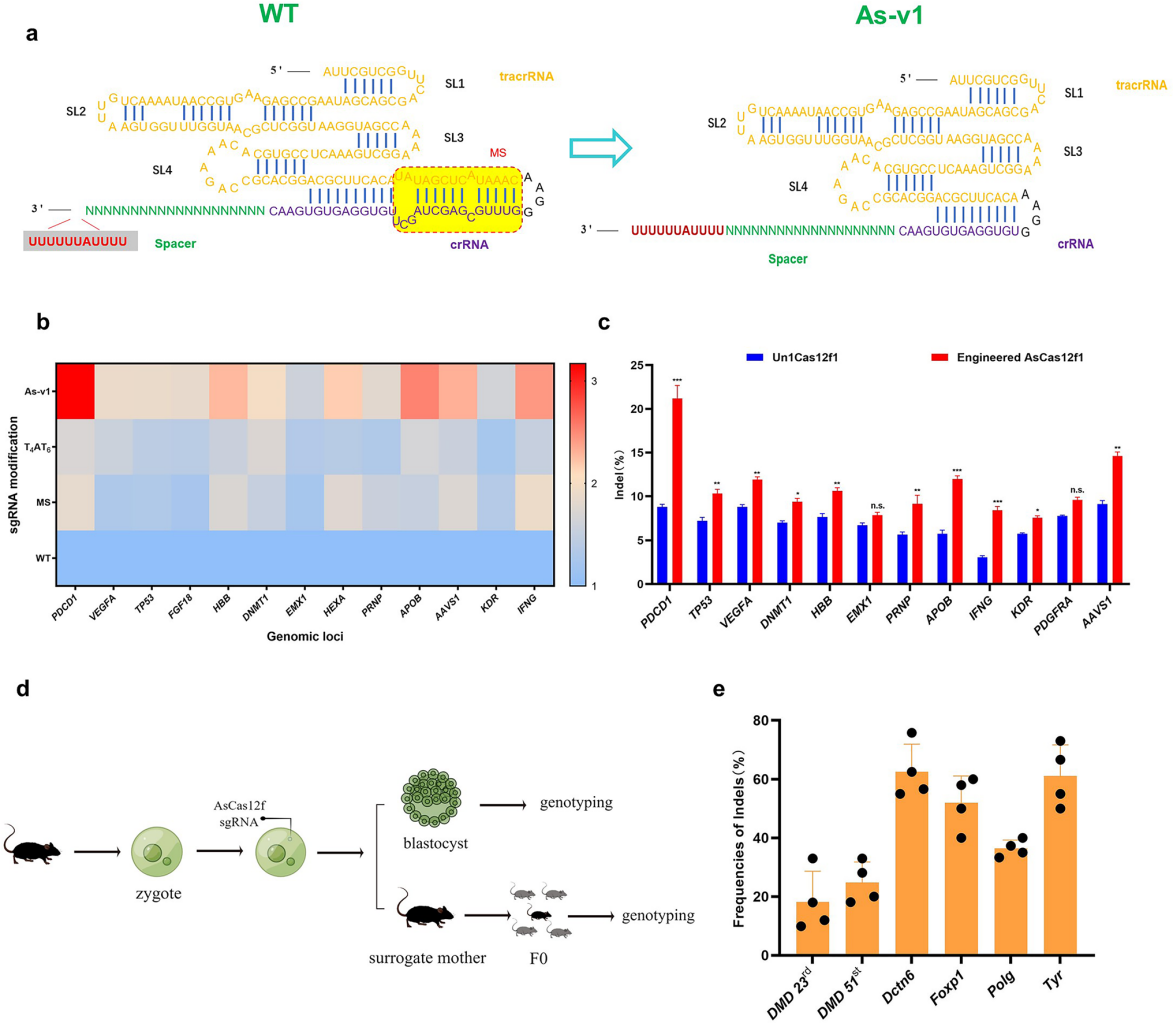
Engineered AsCas12f1 gRNA augments editing frequency. **a** The comparison between AsCas12f1 WT and As-v1 gRNA. The MS is marked in yellow and the T4AT6 is marked in grey. **b** Indel activities of the different modifications of sgRNA with wild-type AsCas12f1 sgRNA (relative to WT) on 13 genomic loci in HEK293T cells. FC, fold change. **c** Comparison of indel editing efficiencies of UnCas12f1 and engineered AsCas12f1 at 12 genomic sites in HEK293T cells. Values and error bars represent mean and s.d. (*n* = 3). *p < 0.05; **p < 0.01; ***p < 0.001. **d** Workflow for genome editing in mouse zygotes or to obtain F0 mice. **e** Average frequencies of indels at six target sites using AsCas12f1 in mouse blastocysts. Values and error bars represent mean and s.d. (*n* = 4)

The sgRNA of AsCas12f1 is a fusion of a 49 nt CRISPR RNA (crRNA) and a 138 nt trans-activated CRISPR RNA (tracrRNA), consisting of 5 stem loops (Figs. 1b and S2) [5, 24]. Notably, AsCas12f1 sgRNA is much longer and adopts a similar structure with Un1Cas12f1 sgRNA (Fig. 1a, b) [5, 25, 26]. Modification of the 3′ poly-uridine (U) and 5`GGG overhanging on gRNAs have been shown to increase gRNA stability, and consequently improve Cas nuclease efficiency [7, 27, 28]. In this study, we separately fused 5′-TTTTATTTTTTT (T4AT6)-3′ and 5′-GGG-3′ sequence to sgRNAs (Fig. 1c). To test the endogenous editing activity, the four target sites *(PDCD1/TP53/VEGFA/FGF18*) were chose in HEK293T cells. The results showed that T4AT6 significantly improved the efficiency in four target sites compared with the original sgRNA, while GGG had no significant improvement at all locus (Fig. 1d). According to the gRNA engineering strategy for Un1Cas12f1 and SpaCas12f1, we designed schemes of MS (modify site throughout the tracrRNA and crRNA) and DS (delete site of stem 2 region in the tracrRNA) in Fig. 1. As shown in Figs. 1e, f and S3, the MS improved editing efficiency at all target sites ranging from 1.21- to 2.07-fold and significantly improved indels at the PDCD1 site. The DS1 and DS2a failed to yield higher editing efficiency at all sites. The DS2b reduced editing frequency at four sites compared with the WT, while it retained large part sequences of the stem-loop 2 (Fig. 1g, h). Then we combined T4AT6 and MS modifications mentioned above to form the new sgRNA version-As-v1 (Fig. 2a). Compared with other modification and WT sgRNA in HEK293T cell, As-v1 observed 3.17-fold more indels than WT sgRNA at PDCD1 loci, significantly improving the CRISPR-AsCas12f1 system indel activity (Figs. 2b and S3). In addition, we tested AsCas12f1 paired with As-v1 and Un1Cas12f1-ge4.1 across 12 genomic loci. The results showed that AsCas12f1 generated higher efficient editing at all target sites than UnCas12f1, ranging from 1.37- to 2.68-fold (Fig. 2c). Collectively, the AsCas12f1 paired with As-v1 (engineered AsCas12f1 system) significantly improved editing efficiency in mammalian cells. The As-v1 version of sgRNA was used for subsequent efficiency assessment in mice*.*

### Engineered AsCas12f1 system enables robust and faithful gene editing in mouse embryos

To evaluate the feasibility and efficiency of the engineered CRISPR-AsCas12f1 system*,* we tested six target sites from five different genes (*Dmd* [29], *Dctn6* [30], *Foxp1* [31], *Polg* [32], and *Tyr* [33]) in mouse embryos. Genome editing was conducted in mouse zygotes using microinjection of AsCas12f1-encoding mRNA and the appropriate sgRNA as previously reported [33]. The mouse zygotes were cultured in vitro to blastocysts after injection and then genotyped (Fig. 2d). Notably, all six target sites showed efficient editing with an average efficiency ranging from 17.7 to 62.5% (Fig. 2e; Table S5).

### Generation of *Tyr* mutant mice using engineered AsCas12f1 system

**Fig. 3:**
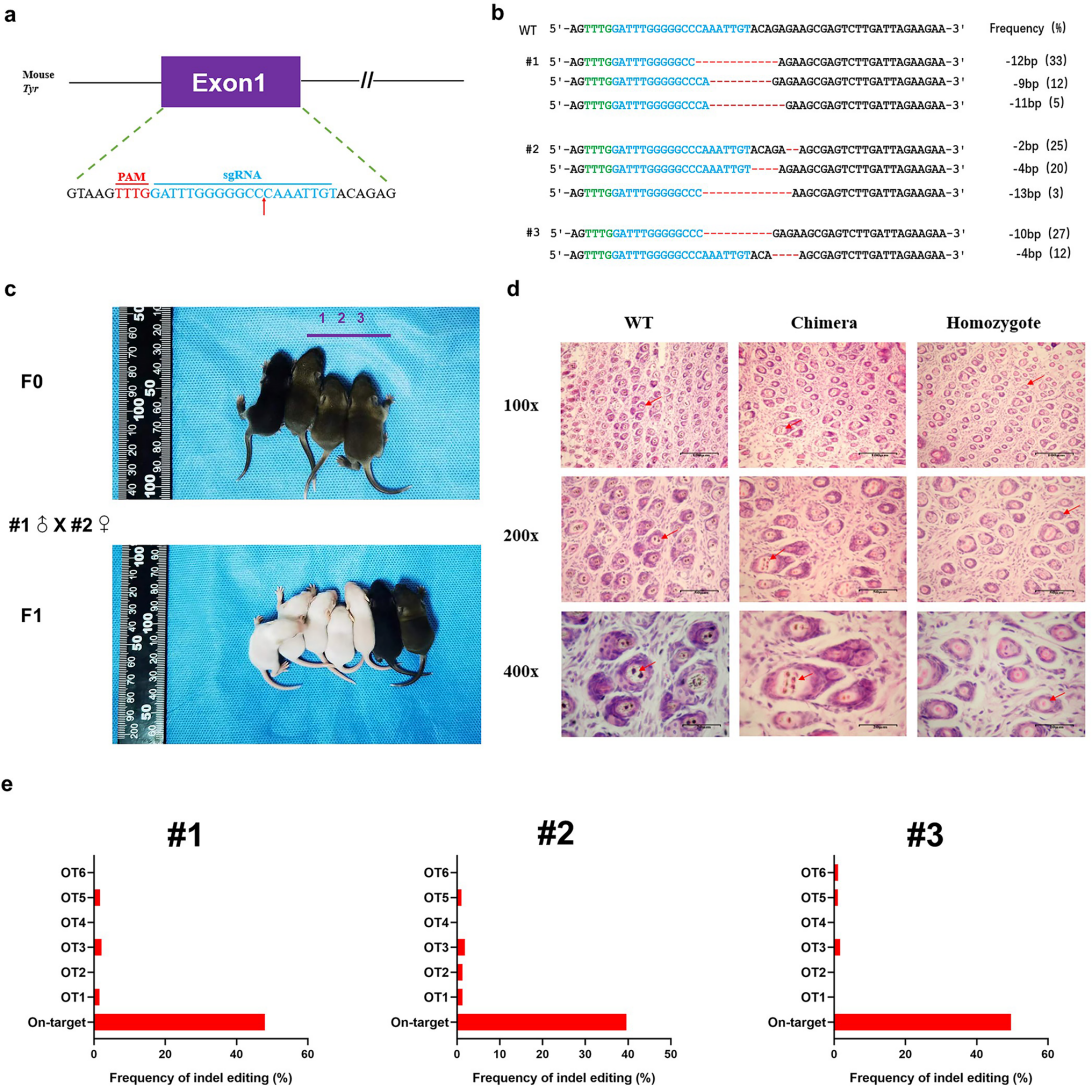
Engineered AsCas12f1 system can induce Tyr mutant mice. **a** The target sequence at the Tyr locus. Target sequence (blue), PAM region (red). The red arrows highlight the cleavage site. **b** Alignments of mutant sequences from targeted deep sequencing. Target sequence (blue), PAM region (green), and indels (red). The column on the right indicates the frequencies of mutant alleles. **c** Photographs of F0 and F1 generation mice generated by engineered AsCas12f1 system. In the F0 generation, three of them showed heterozygous phenotypes with grey complexion. **d** H&E staining of abdominal skin from WT and mutant mice at 100x, 200x, and 400x, respectively. The red arrows highlight the melanin in the basal layer of the epidermis. Scale bars: 100 μm /50 μm/20 μm. H&E, hematoxylin and eosin; WT, wild type. **e** The potential off-target sites (POTs) in Tyr mutant mice were detected by Hi-TOM analysis. All the POTs and primer sequences for the off-target assay are listed in Table S3 and S4

Then we further attempted to examine the practicality of AsCas12f1 on animal disease models. We generated *Tyr* mutant mice to simulate human oculocutaneous albinism (OCA) (Fig. 3a). The *Tyr* mutation is the main cause of OCA, which is characterized by the reduction or absence of melanin in skin, hair, and eyes [34]. After microinjection, we transferred zygotes into surrogate mice. Of the four pups obtained, three contained the expected editing outcomes at the *Tyr* site, with efficiency ranging from 38.6 to 49.7% (Fig. 3b, c). In comparison with the wild-type (WT) mice, they showed heterozygous phenotypes with grey complexion (Fig. 3b). Histological H&E staining further confirmed the local or complete absence of melanin in the skin (Fig. 3d). In addition, deep sequencing revealed that the average frequency of off-target editing in the mutant mice remained low (< 1%) at possible off-target sites (POTs) (Fig. 3e). Since white *Tyr* mutant mice were not available in the F0 generation, we intercrossed the heterozygotes and successfully obtained white mice in the F1 generation (Fig. 3c). All data demonstrated that the mouse model represented the symptoms of the human OCA disease, proving the effectiveness of the AsCas12f1 system in gene editing and mouse model construction.

### Generation of *Dmd* and *Foxp1* mutant mice using the engineered AsCas12f1 system

**Fig. 4:**
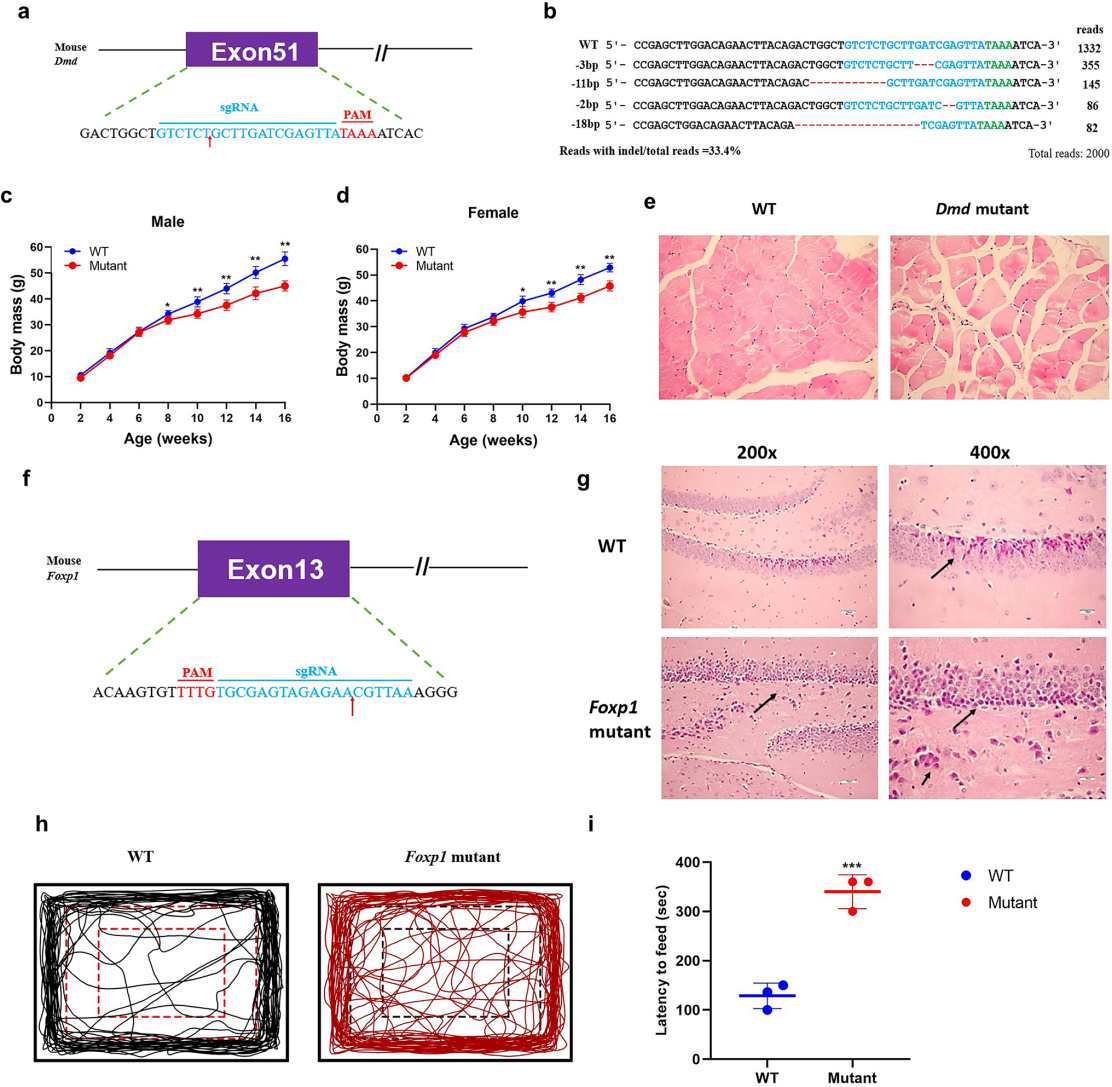
Engineered AsCas12f1 system can induce Dmd and Foxp1 mutant mice. **a** The target sequence at the Dmd locus. Target sequence (blue), PAM region (red). The red arrows highlight the cleavage site. **b** Alignments of mutant sequences from targeted deep sequencing. Target sequence (blue), PAM region (green), and indels (red). The column on the right indicates the frequencies of mutant alleles. **c** Body mass comparison of male Dmd mutant and WT (*n* = 3). **d** Body mass comparison of female Dmd mutant and WT (*n* = 3). **e** H&E staining of gastrocnemius from WT and mutant mice at 200x. Dmd mutant mice displayed myopathy with excessive fiber size variation. **f** The target sequence at the Dmd locus. Target sequence (blue), PAM region (red). The red arrows highlight the cleavage site. **g** H&E staining of hippocampus from WT and mutant mice. (200× and 400×). Foxp1 mutant mice displayed significant nuclear pyknosis (black arrows). **h** Travel path of wild-type mice and Foxp1 mutant mice in the open field. Foxp1 mutant mice displayed a higher activity level within the open field than WT mice. **i** Latency to feed of the mice in the novelty-suppressed feeding assay. Foxp1 mutant mice displayed a lower activity level within the novelty-suppressed feeding than WT mice

In addition, we also attempted to mimic the Duchenne muscular dystrophy (*Dmd)* gene and ***Fork-head Box P subfamily 1 (*Foxp1*) gene mutant mice (Fig. 4). Duchenne muscular dystrophy is caused by mutations in the *Dmd* gene, which encodes dystrophin protein. These mutations cause frameshift and/or premature stop codon formation in the *Dmd* gene, thus disrupting the expression of dystrophin and leading to the development of *Dmd* [29, 35]. Deep sequencing results showed that 34.3% indels occurred at *Dmd* loci in the F0 generation (Fig. 4b). The *Dmd* mutant mice and control littermates were weighed every 2 weeks, and the data showed that both the female and male mutant mice were significantly lighter than their control littermates (Fig. 4c, d). As shown in Fig. 4e, the *Dmd* mutant mouse displayed typical muscular dystrophy signs, as evidenced by increased fiber size variation.

Fork-head Box P subfamily 1 (*Foxp1*) has been linked to neurodevelopmental disorders, suggesting that it may play a central role in various cognitive and social processes [36, 37]. *Foxp1*-specific deletions and mutations have been reported in patients with intellectual disability (ID), autism spectrum disorder (ASD), speech and language deficits [31, 37]. We generated *Foxp1* mutant mice to simulate human *Foxp1* mutations (Fig. 4f). The target deep sequencing results showed that the efficiency ranged from 26.9 to 37.8% (Fig. S4a). The *Foxp1* mutant mice were smaller in size compared with their WT littermates (Fig. S4b). Moreover, histological H&E staining revealed a large number of nuclear pyknosis in *Foxp1* mutant hippocampus (Fig. 4g). Due to hippocampal damage, short-term memory is impaired in *Foxp1* mutant mice. For both distances traveled and total rearing time, *Foxp1* mutant mice displayed a higher activity level within the open field than wild-type mice (Fig. 4h). In the novelty-suppressed feeding assay, the latency of each mouse to approach and eat a new food was used as an index of anxiety-like behavior with longer latency indicating anxiety [38, 39]. As shown in Fig. 4i, the latency to feeding increased sharply in *Foxp1* mutant mice. It revealed that *Foxp1* mutant mice displayed significantly lower anxiety. Furthermore, no mutations were detected at the potential off-target sites in *Dmd* and *Foxp1* mutant mice (Fig. S5a and b), indicating the efficiency and precision of AsCas12f1 system. In conclusion, we validated the feasibility of the CRISPR-AsCas12f1 system in mammals and successfully constructed multiple mouse disease models for therapy.

## Discussion

In this study, we engineered a sgRNA variant for CRISPR-AsCas12f1 system, which can induce genome editing 3.17-fold more efficiently than wild-type sgRNA in mammalian cells. In addition, we confirmed that the engineered AsCas12f1 system showed the feasibility and efficiency in *Tyr/Dmd/Foxp1/Polg* mutant mouse embryos. Notably, this strategy can be used to generate *Tyr/Dmd/Foxp1* mutant mouse disease models in the F0 generation. These results demonstrated that a higher efficiency could be achieved by the engineered AsCas12f1 system in cells and mice.

Cas12f1 has an extra-long gRNA for its compact protein size, which may be associated with Cas12f1’s ssDNA cleavage activity [7]. Through the analysis of sgRNA engineering strategies, we found that some regions do not directly interact with effector proteins. In addition, we tried to truncate parts of original sgRNA to improve dsDNA cleavage activity. It is interesting that our MS approach was in line with recent studies [13, 14], in that the trimmed regions are structurally disordered. One interpretation would be that the flexibility of disordered region might hinder AsCas12f1 homodimerization [7]. Moreover, all DS modifications of stem 2 region resulted in significant decrease in indels, indicating that stem 2 is a major contributor to sgRNA–protein interactions. The T4AT6 method may obviate the termination signal for the U6 promoter and enhance the compatibility between the gRNA and AsCas12f1, thereby improving the editing efficiency [7, 40]. Together, these may be the reasons for the efficiency improvement of our engineered AsCas12f1 system.

Although our engineered AsCas12f1 system has induced stable gene editing in cells and mouse embryos, we observed its editing efficiency could be further improved. Despite of sgRNA engineering, genome editing efficiency can also be improved by structural analysis and protein engineering [4, 13, 14, 41]. With the assistance of structural information, protein residues near the nucleic acid were replaced to introduce more interactions or promote conformational flexibility [13, 41]. The variants with enhanced activity of AsCas12f1 were then further increased by combining beneficial mutations in previous studies, such as AsCas12f1-YHAM (F48Y/S188H/V232A/E316M) [13], AsCas12f-HKRA (I123H/D195K/D208R/V232A) [13], enAsCas12f1 (D196K/N199K/G276R/N328G/D364R) [14], and AsCas12f1-v5.1 (N70Q / K103R /A104R /S118A /D364R) [41]. Although these protein engineering schemes have significantly improved the activity of AsCas12f1 protein [13, 14, 41], different engineering strategies and screening methods could result in different effective mutation combinations. Therefore, the optimal combination of AsCas12f1 protein mutations needs to be verified in the future.

Notably, we successfully generated three mouse mutant models, fully demonstrating the feasibility and stability of the engineered AsCas12f1 system in mice. In comparison with adeno-associated virus (AAV)-based gene delivery [13, 42], our animal models were obtained by microinjection and embryo transfer. The microinjection process, typically visualized under a microscope, allows for real-time monitoring of delivery success.

Taken together, we converted AsCas12f1 into an efficient genome editor by gRNA engineering. This study develops a novel theoretical reference for the engineering modification of CRISPR system, and provides a compact gene editing tool for basic research with remarkable promise for therapeutic applications.

### Supplementary Information

Below is the link to the electronic supplementary material.

Supplementary file1 (DOCX 741 KB)

## Data Availability

The authors state that all data necessary for confirming the conclusions presented in this article are represented fully within the article. The datasets generated and/or analyzed during the current study are available from the author upon reasonable request.
