# Supplementary material for: Targeted mutagenesis in mice via an engineered AsCas12f1 system
Source: Cell Mol Life Sci. 2024 Jan 28;81(1):63. doi: 10.1007/s00018-023-05100-3 (PMC10821844; doi:10.1007/s00018-023-05100-3)
Supplement: Supplementary file 1 — Supplementary file1 (DOCX 741 KB) [file 18_2023_5100_MOESM1_ESM.docx]

**Supplemental file**

**Targeted mutagenesis in mice via an engineered AsCas12f1 system**

Peng Fan**^#^**, HejunWang**^#^**, Feiyu Zhao, Tao Zhang, Jinze Li, Xiaodi Sun, Yongduo Yu，Haoyang Xiong, Liangxue Lai*, Tingting Sui*


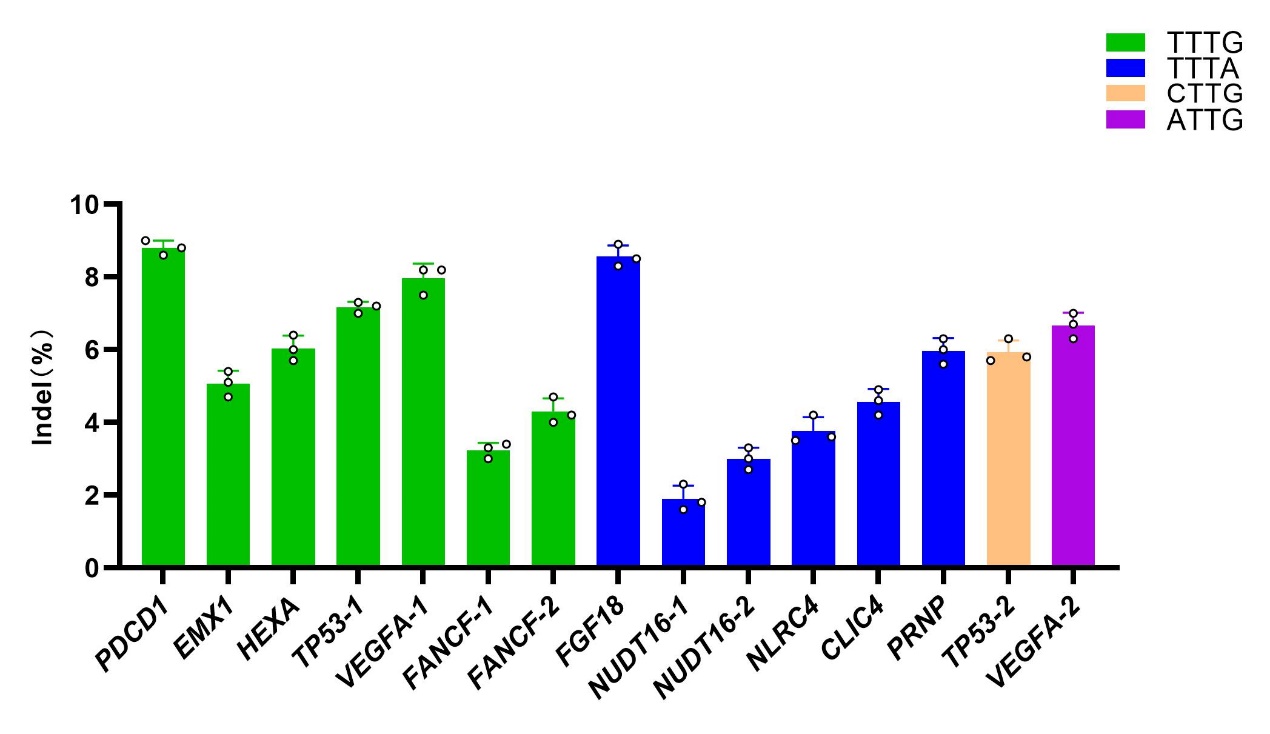


**Fig.S1. Indel efficiencies on 15 endogenous loci in HEK293T cells, determined by NS (next-generation sequencing).** The data are presented as the mean ± s.d. (*n* = 3).

**
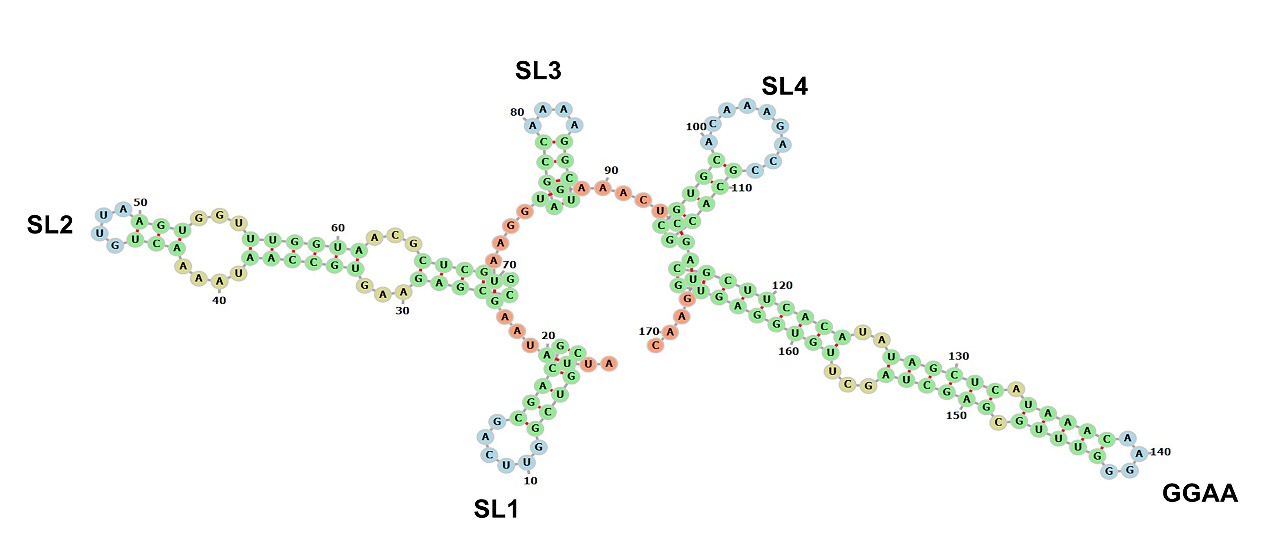
**

**Fig. S2. The predicted Secondary Structure of AsCas12f1 gRNA.** SL, stem loop. GGAA, GGAA loop.


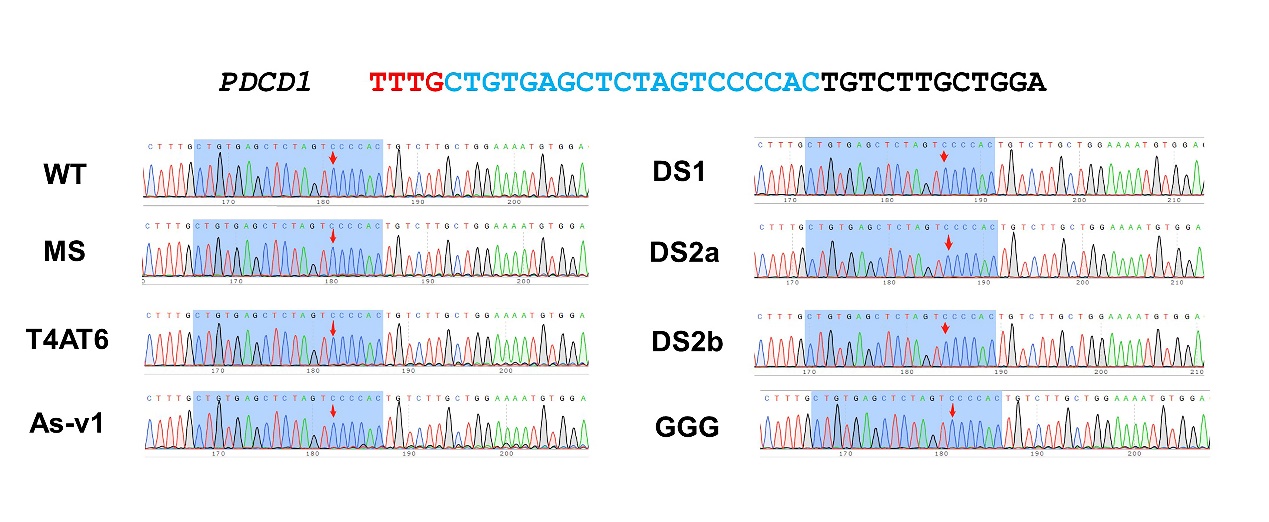


**Fig. S3. Sanger sequencing chromatograms of different gRNA schemes mediated indelsin human HEK293T cells.**

The PAM sequence and spacer sequence of gRNA were highlighted in red and blue, respectively. Red arrows indicate the targeted bases.


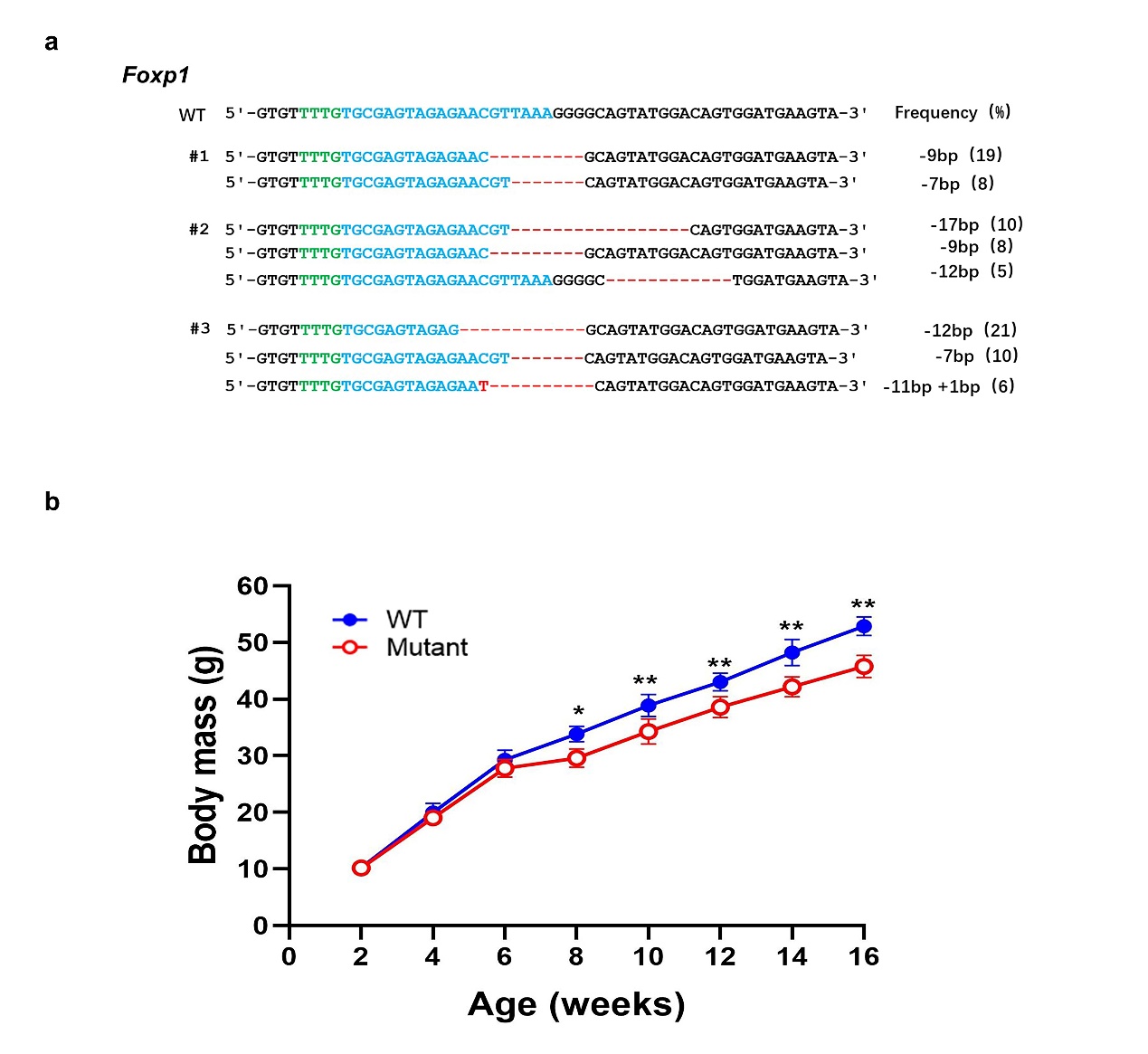


**Fig. S4. Analysis of F0 generation mouse disease models.**

1. Deep sequencing results of *Foxp1* mutant mice. Target sequence (blue), PAM region (green), and indels (red). The column on the right indicates the frequencies of mutant alleles.
2. Body mass comparison of WT and *Foxp1* mutant mice. (*n*= 3).


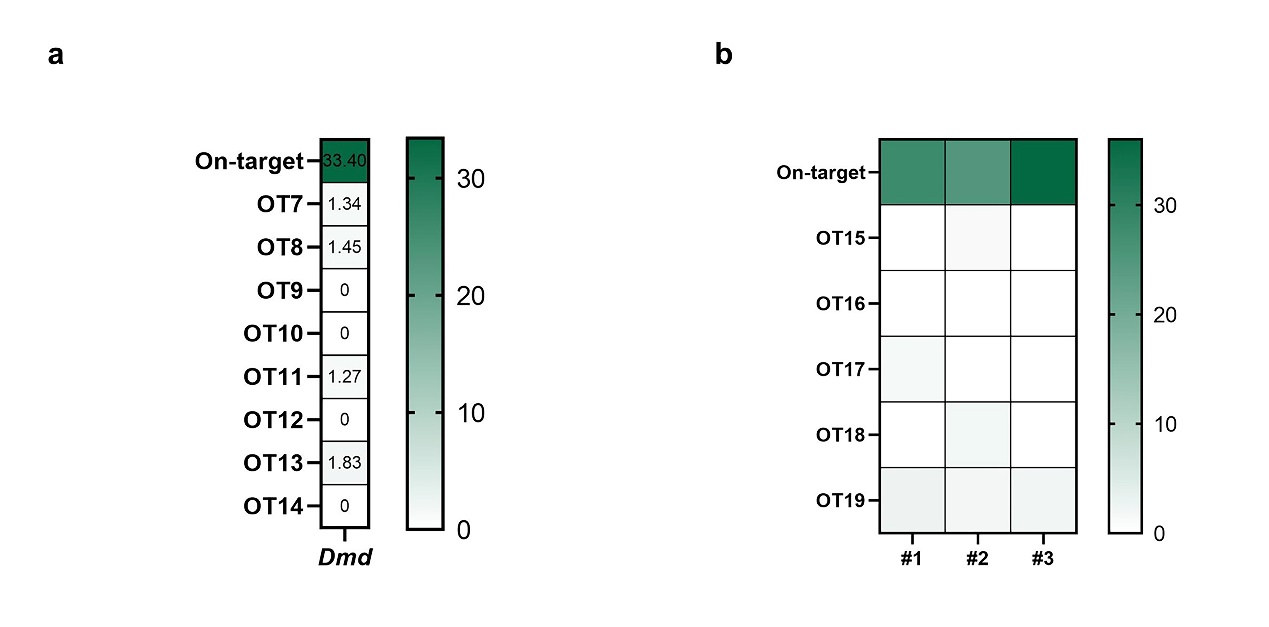


**Fig. S5. Off-target analysis of *Dmd* and *Foxp1* mutant mice were detected by Hi-TOM analysis.**

1. The deep sequencing of potential off-target sites in *Dmd* mutant mouse.
2. The deep sequencing of potential off-target sites in *Foxp1* mutant mice.

**Table S1. Sequences of sgRNA used in HEK293T cells and mice.**

| **Description** | **Gene** | **PAM** | **Sequence** |
| --- | --- | --- | --- |
| **Human**  **HEK293T cells** | *PDCD1* | TTTG/TTTA/  CTTG/ATTG | CTGTGAGCTCTAGTCCCCAC |
|  | *EMX1* |  | TACTTTGTCCTCCGGTTCTG |
|  | *TP53-1*  *VEGFA-1*  *DNMT1*  *HBB1*  *FGF18*  *HEXA*  *PRNP*  *APOB*  *AAVS1*  *KDR*  *IFNG*  *TTR*  *PDGFRA*  *HBB2*  *VEGFA-2*  *CLIC4*  *NUDT16-1*  *NUDT16-2*  *FANCF-1*  *FANCF-2*  *TP53-2*  *NLRC4* |  | AGGCATCACTGCCCCCTGAT  CTCTCAAGACCCACAATCCA  TGTGGCCACAAGGCTCAGTT  GTAGCAATTTGTACTGATGG  AGAACACATACCCCTGGGCC  AGTATACGCTTCCACAGAAA  TGGCCACATGGAGTGACCTG  CTGTCGACACCCAGAATCAT  CCTGGACACCCCGTTCTCCT  GAAACCTGTCCACTTACCTG  CCAGGGCGAAGTGGGGAGGT  ACCATCAGAGGACACTTGGA  CATATCATCCACTCAATATC  TACTGATGGTATGGGGCCAA  AAGAAGGGATGTGGTGCATT  CCCTGGCTACCTCCCCTACC  GGGGTAGAGGTACTCTACAG  GGGGTAGAGGTAGTCTACAG  GGTTCTCTCTATAGCCATTG  ACTTTAGTGACTAGCCGCCA  ATAAGAGGTCCCAAGACTTA  GAGGGAGACACAAGTTGATA |
| **N2a cells**  **and mouse embryos** | *Dmd23 ^rd^* | TTTG | AGGCTCTGCAAAGTTCTTTG |
|  | *Dmd51 ^st^*  *Dctn6*  *Foxp1*  *Polg* | TTTA  TTTG  TTTG  TTTG | TAACTCGATCAAGCAGAGAC  GATTTTTCAAGACAGCCTCC  TGCGAGTAGAGAACGTTAAA  ACCGAGCCCATATCAGGGAA |
|  | *Tyr* | TTTG | GATTTGGGGGCCCAAATTGT |

**Table S2. Primers used for genotyping in HEK293T cells and mice.**

| **Description** | **Forward** | **Reverse** |
| --- | --- | --- |
| *PDCD1* | TGGATCTTAAGCGCCTGTGTTG | CTCTTCCTTCTACGTGAGGCTG |
| *TP53-1*  *TP53-2* | CTTTGAGGTGCGTGTTTGTG  AGGTAAGCAAGCAGGACAAG | GAAAGCTGGTCTGGTCCTTTA  CAGGAAGTAACACCATCGTAAGT |
| *EMX1* | GGCCTCCTGAGTTTCTCATCT | TGTCCCTCTGTCAATGGCG |
| *VEGFA-1*  *VEGFA-2* | GAGCACCTGGCAAGTAGTAAA  CAAAGTGAGTGACCTGCTTTTGG | TAGATGCCAGCTTCCTCAAATC  ACAGCAGAAAGTTCATGGTTTCG |
| *DNMT1*  *HBB*  *FGF18*  *HEXA*  *PRNP*  *APOB*  *AAVS1*  *KDR*  *TTR*  *IFNG*  *NUDT16*  *FANCF*  *PDGFRA*  *CLIC4*  *NLRC4* | CTAGTATCTCGCCAACCTGACC  CATATTCTGGAGACGCAGGAAG  CTCAGACTAGCGCATCTCAAC  GAAGAGTTTTCTGTGGTGTGAGC  AAGTGCTTCAGAGAAGTACAGGG  GGTGCTCACAAGGCGACACTAAGG  TGCCCAAGGATGCTCTTTC  TTACCCCCAAAATGGAATCA  CCAGATTTCTAATACCACAAAG  TCAAGCTGATCAGGTCCAAAG  AGTATAGAAGAGCCAGGTAGGG  GCCTGACAGAGGCTTTGAA  TCTGGGACACGAGCTATTCC  TCCCAGTGATAAGGAGGTTGA  TTCCCACTCCACTTTGTTCC | GTGAAGCCAATGACCACAGAAC  GCCTATCAGAAACCCAAGAGTC  AGACACACAAGAGCCAAATACA  GCCTCTTATAGGAGACTGAAGGC  GTCACTGCCGAAATGTATGATGG  GCCACATGCAGCTTCAGGGGTTC  AGCACAGACTAGAGAGGTAAGG  GCCCCCAGAAGGTAAAAGAG  CAGGTGTCATCAGCAGCCTTTC  ACCTGTGTGGCTTGTATTGT  TCAGGATGAGCAGCACTTTG  GGGATGCAGCTCGTTACC  AGGTGGGCAGATAAGGGACT  AGCTCTCTTCTCTGGTGGTAATA  CGCAGACCCTATTCATGTTCTA |
| *Tyr*  *Dctn6*  *Dmd23^rd^*  *Dmd51^st^* | AGTCTGTGACACTCATTAACCTATT  CTACTCAACACTAGCATCCCTTC  ACTTCTGTGATGTGAGGACATATAA  CTGAGCCACTATGCTTGTAAGT | GGTGTTGACCCATTGTTCATTT  GACTGTGTAAGACTCCTGCTAAC  CAAACCTCGGCTTACCTGAA  GTTCTCTGGTGTACTGCCTTATC |
| *Polg*  *Foxp1*  *IVT-T7* | GTGTGGGTGGTGTGGAATAG  CTTGCTGCAGAATGCAGTG  TAATACGACTCACTATAGGGATTCGTCGGTT | ACCATGCTAGTGTGTTGTAAGT  CGCATGGGATGTAACAGAGA  CTGCCATTTGTCTCGAGGTC |

**Table S3. The potential off-target sites (POTS) used in this study. The mismatched nucleotides are shown in red.**

| **Description** | **Potential Off Target Site** | **Number of mismatches** | **PositionOT** |
| --- | --- | --- | --- |
| OT1  OT2 | GATTTGaGtGaCCAAATTGT  cATTTGaGGGCtCAAATTGT | 3  3 | Chr5: + 124136342  Chr7: +93246882 |
| OT3 | GcTTTGGGGGtCCAAATTtT | 3 | Chr9: ­115964971 |
| OT4 | GATTTGGGGGCCtAAtTTGT | 2 | Chr6: ­63441721 |
| OT5 | GATaTtGGGGCCaAAATTGT | 3 | Chr3: + 181491079 |
| OT6  OT7  OT8  OT9  OT10  OT11  OT12  OT13  OT14  OT15  OT16  OT17  OT18  OT19 | tATTTGGtGGCCCAAAaTGT  TAACTtGATCAAGCAGAGAa  TAACTCaATaAAGCAaAGAC  TAACTaGtTCAAGCAaAGAC  TAACTCaATttAGCAGAGAC  TAACTtGATCcAGCAGtGAC  TAACTCtAgCAAGgAGAGAC  TAACTCaATCAAGCtGAGgC  TAtCTCtATCAAcCAGAGAC  TGCGAGTAGAaAACGTTAAA  TGCcAGTAGAaAACaTTAAA  TGCaAGTAGAGAACGTcAgA  TtCGAGTAGAaAAtGTTAAA  TGaGtGTAGAGAgCaTTAAA | 3  2  3  3  3  3  3  3  3  1  3  3  3  4 | Chr3: + 189380015  ChrX: + 31774020  Chr8: + 122974471  Chr22: ­ 25296024  Chr7: ­ 109364594  Chr2: ­ 164289652  Chr14: ­ 50809472  Chr13: ­ 25624374  Chr4: ­ 146276226  Chr3: ­ 70972616  Chr5: ­ 99569693  Chr1: ­ 199160893  Chr7: + 114662100  Chr1: + 85046018 |

**Table S4. Primers used for identifying off-target assay.**

| **Description** | **Forward** | **Reverse** |
| --- | --- | --- |
| OT1 | TCCTGCCTCAATAAAGACGTATC | AAGAAGATCCAGTTAGCAGAATGA |
| OT2 | CCAAGGCATACGGGAGATAAG | GAGAAGCCAAGACCAGAGATG |
| OT3 | GACACATTTCTGTCATGGGAAC | GTCACAGCATTAGGAAAGTTGAA |
| OT4 | ACAGAGAGAACCACAGGACTTA | GGATTTCTGCCTGAGCTTACTT |
| OT5 | GTGATGTGATCATTGTAGAATTGGA | CCTGAAGGTGACACCATTACT |
| OT6  OT7  OT8  OT9  OT10  OT11  OT12  OT13  OT14  OT15  OT16  OT17  OT18  OT19 | GTTTGGCCAACACTCAAGAATA  AAGACAAAGGGTCCTGCATAC  GTTCCTGTGTCTGTGTGTGT  CACCACCCAACCTCAAGAAA  TTGACCTTACTGAGCAGTTTCC  ACACCAGATGGGATGTCAAAG  GTCTGGAAATCATAGGAGGTAGG  TGGAAGTGGTATATGTGCTAGTG  AGGACAGCAGAGACCTGAA  TGTCACTGGAAGGTTTCTTACC  CACTGAGCACCAGCTAATCTT  AGAGATGGTCTCTACAGGTTCTAT  TTGCTCCTCTTGGCTCTTTAC  TAGTACCAGCCAAGATCAGAGA | AAACAATGGGAGTGAGAATGAC  TCTCCAAAGGGACACACAAAG  CCTTCTCACTCTTGGCTACTTG  GGGCTACATAGAAAGCCAGAAG  TTTCAGAGCACGGAAGAACC  GTACAAGAGCTGCCTTCAGTTA  CCATATTGTAGGGTGTGGAGAG  TGGGAGGACATGAGAGTAGAA  GCAACAAAGTAGCGGATGAAAG  CAACACAACAGGCTCAATCTTT  GGCTATTCCTGGACCACTTTAC  GGAGAGAGGAGAAGAGGGTAAA  CACAAGCACTCACTCTTCCA  CATCAGGAGCGAGTTAGTCAAG |

**Table S5. Summary of embryonic development and frequency of gene editing.**

| **Target**  **Site** | **No.of**  **zygetos** | **No.of**  **blastocysts** | **Mutation ratio**  **（no.of mutated/**  **total blastocysts）** |
| --- | --- | --- | --- |
| *Tyr*  *Dmd23 ^rd^*  *Dmd51 ^st^*  *Dctn6*  *Foxp1*  *Polg* | 11  17  16  12  18  18 | 7（63.4%）  12（70.6%）  10（62.5%）  8（75%）  12（66.7%）  12（66.7%） | 4  6  4  5  4  7 |
